# Langerhans Cell Histiocytosis Presenting as a Limp in a Child: A Case Report

**DOI:** 10.7759/cureus.83149

**Published:** 2025-04-28

**Authors:** Eric Chun-Pu Chu, Cliff Tao, Gordon Cheung

**Affiliations:** 1 Chiropractic and Physiotherapy Centre, New York Medical Group, Hong Kong, CHN; 2 Chiropractic Radiology, Private Practice, Irvine, USA

**Keywords:** braf v600e mutation, childhood cancer, langerhans cell histiocytosis, multifocal bone disease, musculoskeletal oncology, osteolytic lesions, pathological fracture, pediatric limping

## Abstract

We report a case of multifocal Langerhans cell histiocytosis (LCH) in a previously healthy four-year-old male child who initially presented to the chiropractic clinic with a three-week history of progressive left-sided limping and nocturnal leg pain. Upon evaluation, the patient demonstrated an antalgic gait, point tenderness over the left femur and tibia, and constitutional symptoms including low-grade fevers, fatigue, and recent weight loss. Radiographic evaluation revealed multiple lesions in the left femur, tibia, and pelvis, with subsequent MRI confirming infiltrative lesions and a pathological fracture of the femoral neck. PET scan identified additional lesions in the skull, spine, and right humerus with no visceral involvement. Biopsy of the femoral lesion confirmed LCH with CD1a and CD207 (langerin) positivity and BRAF V600E mutation. The patient was classified as having multifocal bone disease without risk of organ involvement, and treatment was commenced with vinblastine and prednisone per standard protocol. This case highlights the importance of recognizing red flag symptoms in pediatric limping presentations, demonstrates the critical role played by non-oncology providers in early detection of serious pathology, and illustrates the characteristic radiographic and histopathological features of multifocal LCH. The case further emphasizes how recent advances in understanding LCH's molecular pathogenesis as a clonal neoplastic disorder driven by mitogen-activated protein kinase (MAPK) mutations have refined our approach to the diagnosis and treatment of this rare but significant pediatric condition.

## Introduction

Langerhans cell histiocytosis (LCH) is a rare myeloid neoplastic disorder characterized by the abnormal proliferation and accumulation of dendritic cells bearing the CD1a+/CD207+ phenotype. With an annual incidence of 4.6 cases per million children under 15 years of age and a slight male predominance (male-to-female ratio of 1.2:1), LCH represents one of the more common pediatric histiocytic disorders [1]. The clinical presentation of LCH spans a remarkably heterogeneous spectrum, from isolated bone lesions with excellent prognosis to aggressive multisystem disease involving high-risk organs (liver, spleen, or bone marrow), which carries significant morbidity and mortality. Recent advances in molecular pathogenesis have identified activating mutations in the mitogen-activated protein kinase (MAPK) pathway, most notably *BRAF* V600E (present in approximately 60% of cases), establishing LCH as a clonal neoplastic process rather than a primary inflammatory disorder as historically considered [1].

The diagnosis of LCH presents significant challenges due to its protean manifestations and nonspecific early symptoms, often leading to diagnostic delays that may impact outcomes [2]. Children frequently present with constitutional symptoms such as fever, fatigue, and weight loss, accompanying more localized complaints including bone pain, skin rashes, or persistent otitis [1]. Bone involvement, present in approximately 80% of pediatric LCH cases, commonly manifests as unexplained limping, focal tenderness, or pathological fractures [3]. These presentations are frequently attributed to more common childhood conditions, with the diagnosis of LCH considered only after failure of conventional treatments or progression of symptoms. Many primary care physicians may be unable to recognize the pattern of LCH as the lead clue for diagnosis [2].

The recognition of red flag symptoms suggestive of LCH is critical across all healthcare disciplines, particularly for providers who may serve as first points of contact for these patients. Key warning signs warranting further investigation include persistent bone pain without clear trauma, progressive limping, unexplained constitutional symptoms, treatment-resistant skin or scalp lesions, and multisystem involvement. Early recognition by non-oncology specialists, including chiropractors, can facilitate prompt referral for appropriate diagnostic imaging and biopsy [4,5]. To the best of our knowledge, this is the first case report of multifocal LCH in a four-year-old child presenting with progressive limping and constitutional symptoms. By highlighting the presentation, diagnostic approach, and management of this case, we aim to increase awareness of LCH's varied presentations among healthcare professionals, emphasize the importance of recognizing concerning patterns of symptoms that warrant advanced imaging, and reduce diagnostic delays that may impact long-term outcomes for children with this challenging disorder.

## Case presentation

A four-year-old boy presented to the chiropractic clinic with a three-week history of progressive right leg limping without any known traumatic event. According to his parents, the limp developed insidiously and was accompanied by significant pain in the right thigh and leg. The pain would occasionally disrupt the child's sleep quality. Over the preceding two months, the parents reported recurring low-grade fevers (typically 38.2-38.7°C), profound fatigue, and a weight loss of approximately 1 kg. The child exhibited notable bone pain and swelling, particularly in the right lower extremity, that had progressively worsened despite rest and over-the-counter analgesics. The parents noted concerning behavioral changes, including increased irritability and a substantial decrease in the child's typical activity level. The family history was non-contributory, with no genetic disorders or similar musculoskeletal conditions reported in first-degree relatives.

Upon presentation, the child appeared generally unwell with visible pallor and demonstrated obvious discomfort with movement of the right lower extremity. Physical examination revealed a pronounced antalgic gait with reduced weight-bearing on the right lower extremity. Inspection of the right leg demonstrated moderate swelling around the distal femur and proximal tibia without accompanying erythema or increased warmth. Palpation elicited marked tenderness, particularly pronounced at the distal femur and proximal tibia regions. Range of motion assessment showed significant limitations in the right hip, with internal rotation reduced to 25° (normal 45°), external rotation to 30° (normal 45°), and flexion to 100° (normal 125°), all limited by pain. Right knee examination revealed extension lacking 10° from full extension and flexion limited to 110° (normal 135°). The right femoral head was notably tender on gentle palpation with increased pain on minimal passive movement. The neurological examination showed intact motor function with 5/5 strength in all extremities except right hip flexion and knee extension, which were reduced to 4/5 due to pain limitation rather than true weakness. Sensory testing and reflexes were unremarkable. Given the concerning presentation of non-traumatic limping with localized bone pain in a young child, the chiropractor recommended immediate advanced diagnostic imaging.

**Figure 1:**
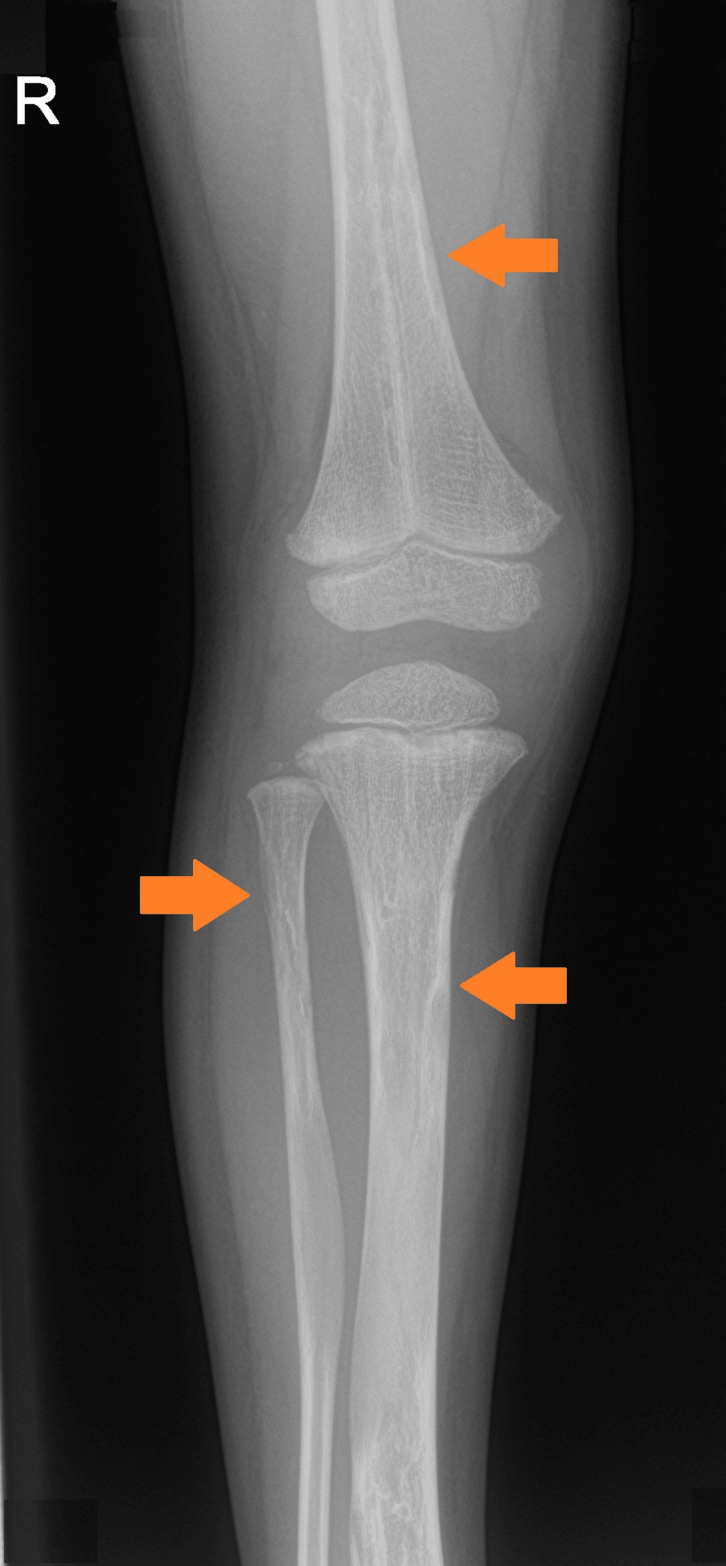
Radiograph of the right knee Multiple mixed-density lesions of the distal femur, and proximal tibia and fibula (orange arrows).

**Figure 2:**
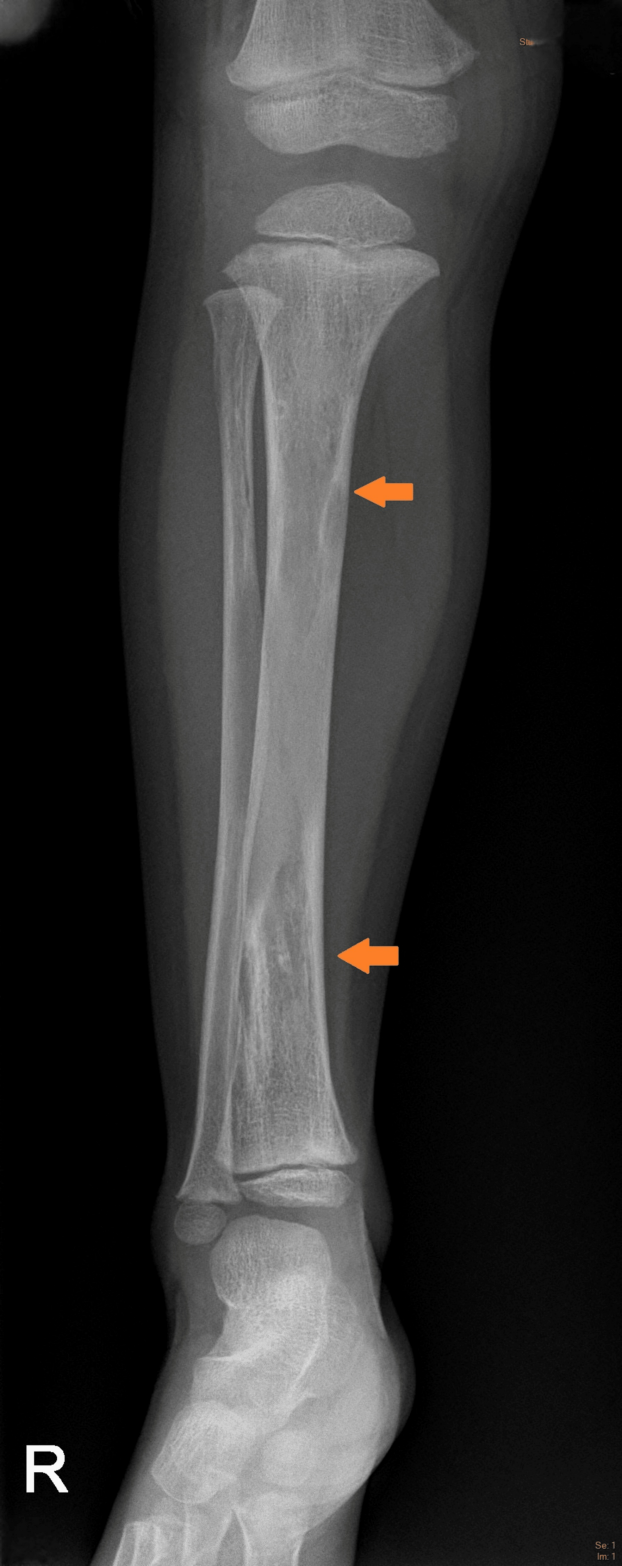
Radiograph of right leg Mixed-density lesions of the tibia and fibula with mild expansion (orange arrows).

**Figure 3:**
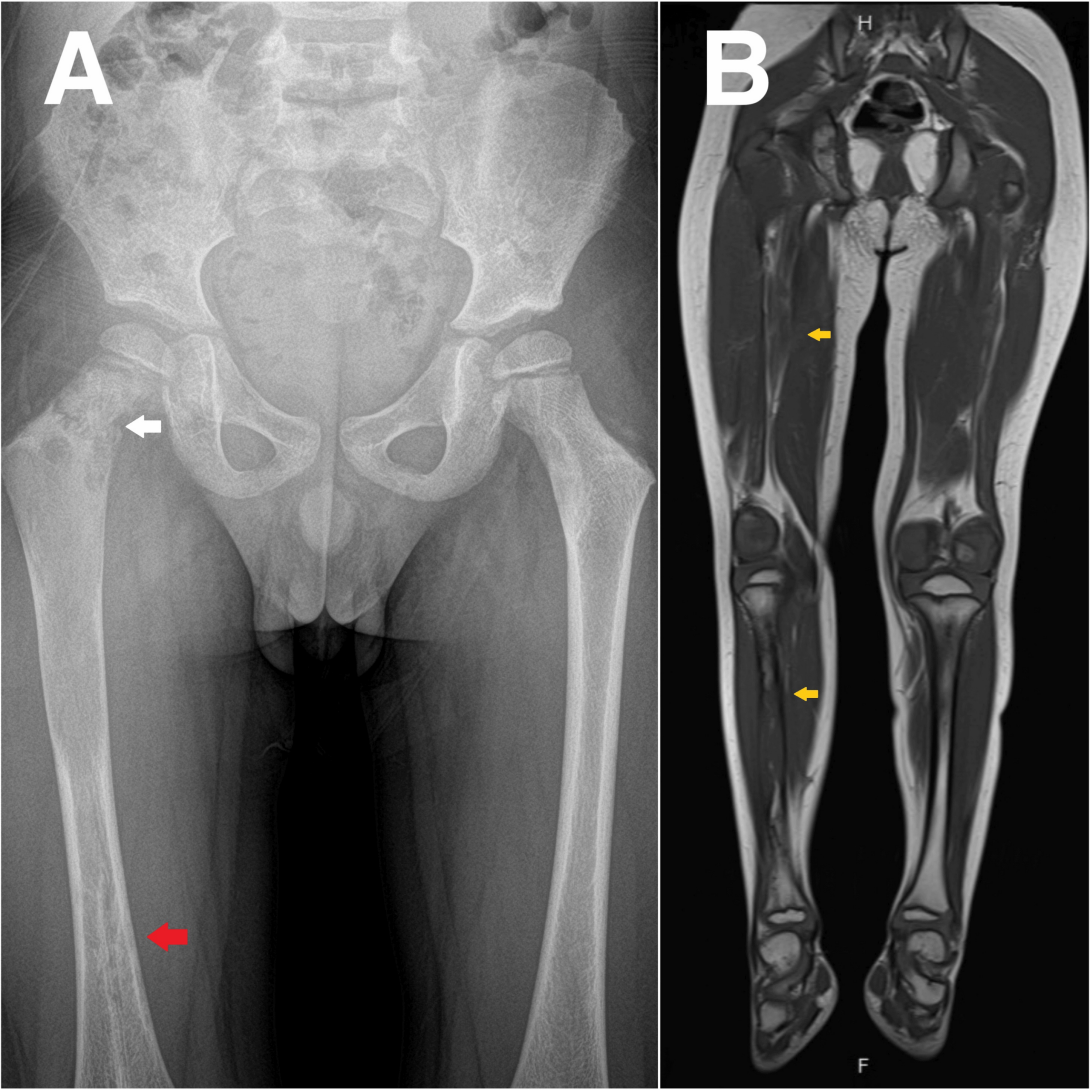
(A) Radiograph of the pelvis and (B) MRI of the lower extremities (A) Pathologic right femoral neck fracture through expansile lesion (white arrow) and sclerotic lesion in the distal right femur (red arrow); (B) Multiple infiltrative marrow intramedullary lesions of the tibiae (yellow arrows).

**Figure 4:**
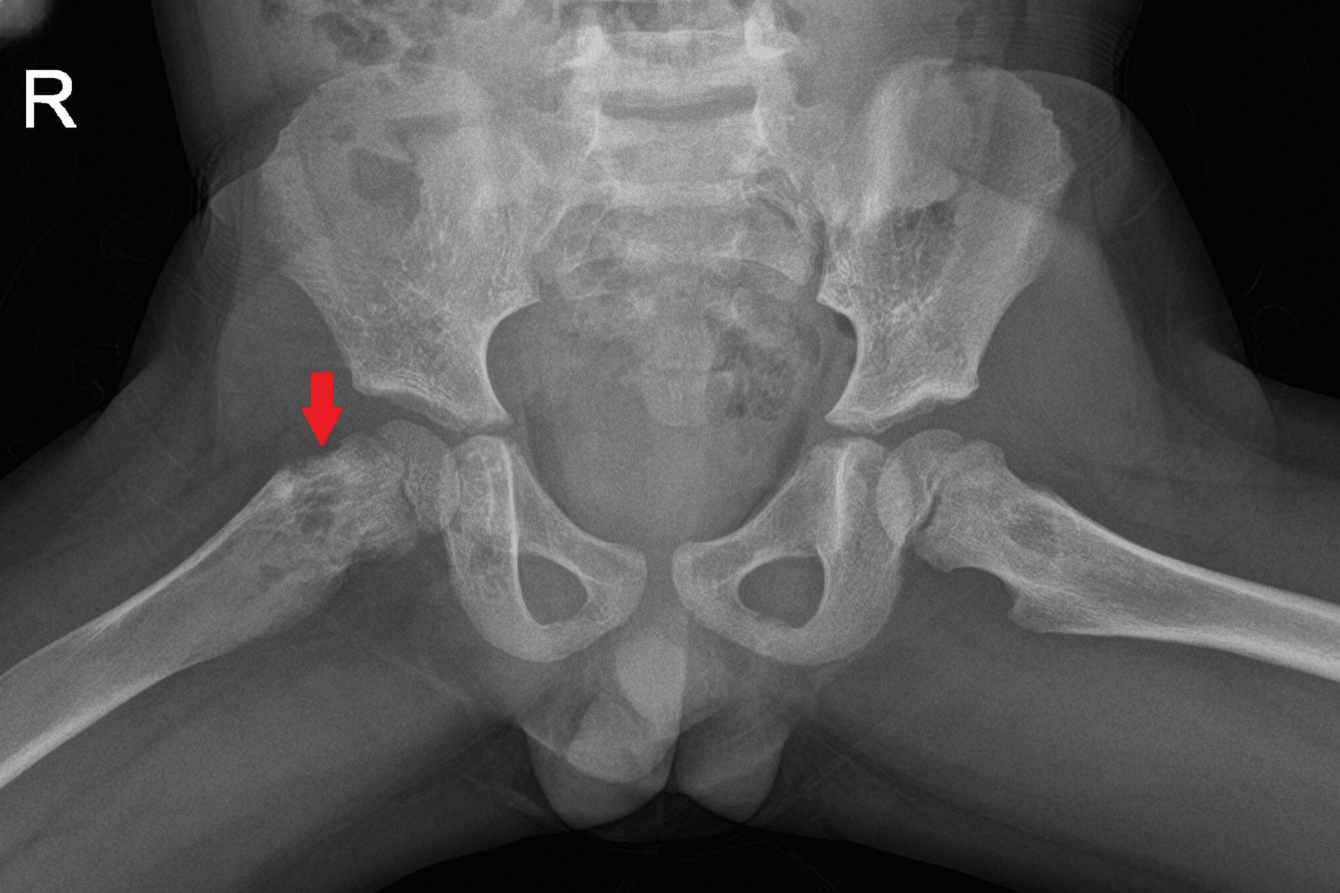
Radiograph of pelvis Pathologic femoral neck fracture through an expansile, mixed density lesion.

Radiographs of the lower extremities revealed multiple variable mixed density lesions, some with mild cortical expansion, most prominently at the right tibia (Figure 1, 2) and distal right femur (Figure 3A). Most concerning was a pathological fracture of the right femoral neck through an expansile bone lesion (Figure 4). Subsequent MRI confirmed these findings and identified extensive infiltrative marrow lesions of the femurs, ischia, right iliac bone, left inferior pubic ramus, tibiae, right fibula, and right tarsal bones, with surrounding marrow edema but no associated soft tissue masses (Figure 3B). 

Laboratory evaluation demonstrated several significant abnormalities, including low creatinine at 0.31 mg/dL (normal range: 0.4-0.6 mg/dL), elevated lactate dehydrogenase at 242 U/L (normal range: 125-220 U/L), and increased C-reactive protein at 5.81 mg/L (normal range: 0-5 mg/L). Complete blood count remained within normal parameters, and calcium and phosphorus levels were unremarkable at 9.4 mg/dL and 4.8 mg/dL, respectively. Alkaline phosphatase was 195 U/L, within the normal range for age (150-380 U/L). The erythrocyte sedimentation rate was mildly elevated at 18 mm/hr (normal: 0-15 mm/hour), consistent with an inflammatory process.

Based on the clinical presentation and examination findings, the favored diagnosis was LCH, prompting immediate referral to pediatric oncology. The patient was advised to minimize weight-bearing on the affected limb and avoid high-impact activities while awaiting specialist evaluation. The pediatric oncology service promptly conducted additional diagnostic studies, including a PET scan, which confirmed the multiple skeletal lesions identified on previous imaging but revealed no evidence of visceral organ or bone marrow involvement. A bone biopsy of the right distal femoral lesion was performed under conscious sedation, with histopathological examination confirming the diagnosis of LCH with CD1a and CD207 (langerin) positivity and *BRAF* V600E mutation. Based on these findings, the oncologist classified the condition as multifocal bone LCH without risk of organ involvement. Treatment is ongoing at the time of publication and consists of a standardized chemotherapy protocol with close monitoring for disease response and potential complications, with early indicators suggesting a favorable response.

## Discussion

This case highlights the critical importance of recognizing warning signs that may herald serious underlying pathology in children presenting with a limp. Non-traumatic limping with progressive worsening, significant pain disrupting sleep, constitutional symptoms (recurring fevers, fatigue, weight loss), and behavioral changes represent a constellation of red flags that should trigger immediate concern. In this patient, the pronounced antalgic gait, localized bone tenderness, and limited range of motion in the absence of trauma appropriately prompted advanced imaging, revealing the characteristic multifocal mixed-density lesions of LCH. Current pediatric guidelines emphasize that the combination of nocturnal bone pain and systemic symptoms carries high sensitivity for serious underlying pathology [6].

This case exemplifies the pivotal role that non-oncology healthcare providers, including chiropractors, play in the initial detection of serious disease in children [7]. As primary healthcare practitioners for musculoskeletal complaints, allied health professionals must maintain vigilance for non-mechanical causes of symptoms. The diagnostic pathway in this case demonstrates an appropriate stepwise approach-recognition of concerning features, immediate advanced imaging rather than conservative management, and prompt referral to pediatric oncology upon identification of suspicious radiographic findings [8]. This sequence aligns with best practice guidelines for suspected bone malignancies in children and illustrates the effectiveness of timely referral networks.

The impact of early recognition on patient outcomes is particularly significant in conditions like LCH, where disease extent and organ involvement influence prognosis. Delayed diagnosis of multifocal LCH increases the risk of progression to high-risk classification and development of permanent sequelae, including neurodegenerative complications. This case reinforces several practice recommendations: thorough assessment of all children with limping, with particular attention to duration, progression, pain patterns, and constitutional symptoms; immediate advanced imaging for non-traumatic limping with any red flag features; development of expedited referral pathways between community practitioners and pediatric specialty care; and implementation of continuing education on recognizing serious underlying conditions presenting with common symptoms like limping. The vigilance and appropriate action demonstrated in this case facilitated timely diagnosis, potentially preventing progression to more extensive disease involvement.

## Conclusions

This report of LCH presenting as progressive limping in a four-year-old child underscores three critical messages for the broader medical community. First, it highlights the importance of recognizing red flags such as progressive, non-traumatic limping, sleep-disrupting bone pain, and constitutional symptoms as potential indicators of serious underlying pathology requiring urgent investigation rather than conservative management. Second, it demonstrates that the role of non-oncology healthcare providers is valuable, and with appropriate vigilance and prompt referral, they can facilitate early diagnosis of rare but significant conditions like LCH. Finally, it reinforces the effectiveness of multidisciplinary care pathways from initial presentation through diagnosis to specialized treatment, emphasizing how timely recognition and appropriate imaging can lead to favorable outcomes even in cases with extensive skeletal involvement. As the understanding of the molecular pathogenesis of LCH evolves from a disorder of uncertain origin to a clearly defined myeloid neoplasm driven by MAPK pathway mutations, this case serves as a reminder that early clinical suspicion and adherence to systematic diagnostic approaches remain fundamental to improving outcomes for children with this challenging condition.
